# The Potential Therapeutic Effects of Platelet-Derived Biomaterials on Osteoporosis: A Comprehensive Review of Current Evidence

**DOI:** 10.1155/2023/9980349

**Published:** 2023-12-07

**Authors:** Mohammad Amin Amiri, Nima Farshidfar, Richard J. Miron, Arkadiusz Dziedzic, Shahram Hamedani, Sajad Daneshi, Lobat Tayebi

**Affiliations:** ^1^Student Research Committee, Shiraz University of Medical Sciences, Shiraz, Iran; ^2^Stem Cells Technology Research Center, Shiraz University of Medical Sciences, Shiraz, Iran; ^3^Department of Periodontology, University of Bern, Bern, Switzerland; ^4^Department of Conservative Dentistry with Endodontics, Medical University of Silesia, Katowice, Poland; ^5^Oral and Dental Disease Research Center, School of Dentistry, Shiraz University of Medical Sciences, Shiraz, Iran; ^6^Marquette University School of Dentistry, Milwaukee, WI 53233, USA

## Abstract

Osteoporosis is a chronic multifactorial condition that affects the skeletal system, leading to the deterioration of bone microstructure and an increased risk of bone fracture. Platelet-derived biomaterials (PDBs), so-called platelet concentrates, such as platelet-rich plasma (PRP) and platelet-rich fibrin (PRF), have shown potential for improving bone healing by addressing microstructural impairment. While the administration of platelet concentrates has yielded positive results in bone regeneration, the optimal method for its administration in the clinical setting is still debatable. This comprehensive review aims to explore the systemic and local use of PRP/PRF for treating various bone defects and acute fractures in patients with osteoporosis. Furthermore, combining PRP/PRF with stem cells or osteoinductive and osteoconductive biomaterials has shown promise in restoring bone microstructural properties, treating bony defects, and improving implant osseointegration in osteoporotic animal models. Here, reviewing the results of in vitro and in vivo studies, this comprehensive evaluation provides a detailed mechanism for how platelet concentrates may support the healing process of osteoporotic bone fractures.

## 1. Introduction

**Figure 1:**
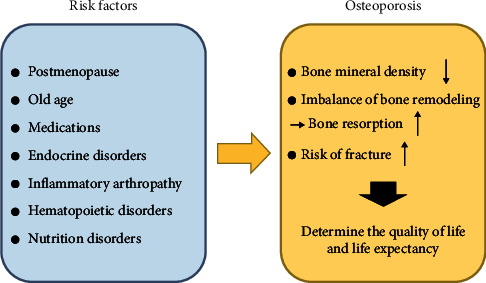
Risk factors for osteoporosis can cause an imbalance in the remodeling process, leading to osteoporosis. Reproduce from reference [7]. Copyright 2020, MDPI, licensed under the terms of the Creative Commons Attribution License (CC BY).

Osteoporosis is a systemic metabolic disorder causing a decrease in bone mineral density (BMD) and microstructural impairment [1]. According to the International Osteoporosis Foundation, 1 out of 3 women and 1 out of 5 men over the age of 50 are suffering from osteoporosis [2]. Moreover, in a meta-analysis by Salari et al., the global prevalence of osteoporosis is estimated at 18.3% worldwide [3]. The current diagnostic criteria for osteoporosis are determined based on BMD which is estimated by a T-score less than or equal to −2.5 in the total hip, femur neck, and lumbar spine [4, 5]. Consequently, a decreased BMD in the skeletal system is associated with a higher incidence of fracture [6]; however, a fraction of bone injuries due to the fragility in osteoporotic patients are reported with BMD values above the mentioned threshold [1]. Aside from the decrease in BMD, many complications are associated with osteoporotic patients.  Figure 1 is also provided to better present the risk factors and common complications of osteoporosis.

The mechanism of osteoporosis is caused by a disbalance in the bone remodeling cycle with more resorption which results in trabecular bone loss, thinning in the cancellous bone, reduced cortical thickness, and enhanced porosity [1]. These processes stem from a shift from osteogenic differentiation towards adipogenic differentiation [8–10]; thus, fewer osteoblasts are produced compared to osteoclasts which impairs the remodeling balance [11]. The transdifferentiation process from osteogenic to adipogenic differentiation is mediated by the activation of adipogenesis genes, including peroxisome proliferator-activated receptor-gamma (PPAR-*γ*) [7]. Studies have also indicated a possible role of PPAR-*γ* in the enhancement of osteoclastic activation through the receptor activator nuclear factor kappa B ligand (RANKL) signaling pathway [7, 12]. These processes can result in impaired biomechanical resistance and bone fragility [1, 13–15].

Among the pharmacological treatments approved by the FDA, bisphosphonates are one of the main choices used to reduce the risk of spine and hip fracture by approximately 50% [15]. However, considerable adverse effects have also been reported for bisphosphonates, therapy, including osteonecrosis of the jaw, difficulty in swallowing, esophageal inflammation, stomach pain, and renal dysfunction [15–17]. Moreover, other pharmacologic interventions, despite their effectiveness in minimizing the effects of osteoporosis on bone structure, have side effects that should be considered before beginning their intervention, such as biliary issues, myocardial infarction, deep vein thrombosis, muscle spasm, nausea, diarrhea, etc. [15].

Therefore, although the current medications have exerted satisfactory results, their adverse effects have encouraged researchers to seek novel approaches for the treatment of osteoporosis and its related fractures. Since osteoporosis management should be based on tissue regeneration principles [18–20], there is growing evidence that the application of platelet-derived biomaterials (PDBs), so-called platelet concentrates, may support a treatment for osteoporosis-related injury [21–24].

In light of novel therapeutic methods utilized in osteoporosis management, the aim of this review article was to evaluate the current evidence based on existing literature concerning the therapeutic effect of PDBs on osteoporosis treatment. The second goal was to provide new insights to further enhance the efficacy of these approaches for future clinical applications, with a main focus on the most recent advancements, possible challenges, and future prospects.

## 2. PDBs Used in Clinical Therapies

Nowadays, PDBs are a promising treatment option in regenerative medicine strategies with primary research focusing on their development and improvements [25, 26]. Platelet concentrates can easily be obtained by centrifugation of the patient's blood collected chairside [27]. These products are highly utilized in regenerative dentistry [28–30], orthopedics [31], dermatology [32], etc. From their cellular and molecular perspective, the high effectiveness of these biomaterials is attributed to their high content of growth factors, cytokines, and regenerative cells collected in supraphysiological doses following centrifugation [33]. Both their high bioactive content and their gradual release of growth factors over time have made them valuable additions to surgical protocols either when utilized alone or combined with other biomaterials [33].

**Figure 2:**
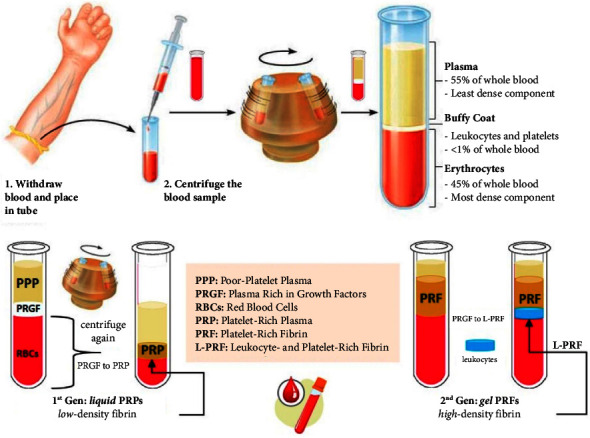
A schematic illustration regarding the preparation of 1^st^ and 2^nd^ generations of PDBs (PRP and PRF). Adapted from [52]. Copyright 2018, MDPI, licensed under the terms of the Creative Commons Attribution License (CC BY).

PDBs are classified based on their preparation protocol and whether or not an anticoagulant is added during their preparation [29]. The first generation of platelet concentrates is termed platelet-rich plasma (PRP) which includes the addition of an anticoagulant to the blood sample which prevents clotting during the centrifugation cycle [34]. PRP is the most widely studied platelet concentrate in regenerative medicine with many investigations demonstrating its benefit in cardiac surgery [35], osteoarthritis [36], osteoporosis [37], dermal rejuvenation [34], and dentistry [38, 39]. It possesses antimicrobial [40], anti-inflammatory [41], and regenerative properties [42]. A second generation platelet concentrate was later termed platelet-rich fibrin (PRF) which was developed with the aim of removing the anticoagulant to favor better healing (Since clotting is one of the first steps to healing) [28, 43, 44]. Unlike PRP which remains liquid in nature, PRF forms a dense fibrin network with cell and growth factor entrapment [45–47]. This feature aids PRF in trapping and releasing bioactive agents over a 2-week period while the fibrin network is more slowly being degraded [45–47]. Studies have also shown that the release pattern of growth factors can differ significantly between these two biomaterials [30, 48]. As it is shown, PRP exerts a burst of release in growth factors during the first 8 h after preparation [30]. However, PRF exhibits a gradual release of growth factors in a 10–28 day period [30, 48]. This difference in the release of growth factors has resulted in the better performance of PRF in inducing cell proliferation and mineralization of osteoblasts [48]. PRF is also demonstrated to excel in neovascularization and wound healing when compared to PRP [49]. From a clinical standpoint, the solid form of PRF has made its application feasible in the areas where the liquid form of PRP cannot be applied [50]. Moreover, some studies have shown that by squeezing the PRF membrane, a liquid exudate is obtained which is known as PRF releasate (PRFr) and has some additional regenerative and antibacterial properties [51]. The fabrication process of PRP and PRF is schematically illustrated in Figure 2 and briefly highlighted in Table 1.

In 2017, the low-speed centrifugation concept (LSCC) was introduced [54]. According to this concept, by modifying the speed and time of centrifugation, the final PRF matrix was more highly concentrated in cells and growth factors. Based on the LSCC concept, a new form of PRF named injectable-PRF (I-PRF) was further introduced [27]. Following centrifugation, liquid-PRF (which should theoretically be known as liquid fibrinogen and thrombin) has yet to form a stable fibrin clot and can be injective similar to PRP, yet is more biologically active [27]. This biomaterial is increasingly utilized in many fields of medicine and dentistry owing to its numerous advantages [27, 55].

## 3. Role of PDBs in Osteoporosis

**Figure 3:**
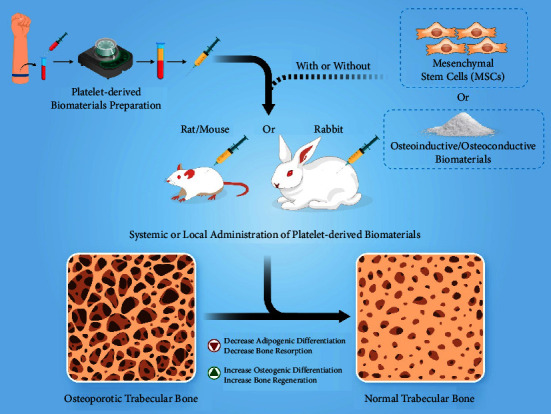
Schematic figure of the preparation method of PDBs and their therapeutic effects on osteoporosis.

Several studies have demonstrated the effectiveness of platelet concentrates for the treatment and management of osteoporosis (Tables 2 and 3) [21–23, 37, 56–64]. All studies were conducted on ovariectomized (OVX) animal models including mice [22, 23, 37, 56], rats [21, 57–60, 62, 64], and rabbits [61, 63]. In these studies, the systemic and localized application of platelet concentrates (PRP or PRF) alone or in combination with osteoinductive and osteoconductive biomaterials has been investigated [21, 37, 57, 58, 60–64]. Furthermore, the additional use of mesenchymal stem cells (MSCs) has also been studied [22, 23, 56, 59].  Figure 3 represents a schematic illustration in this regard. In the following sections, the outcome of both systemic [22, 23, 37, 56] and localized [21, 57–64] administration of platelet concentrates is discussed (Tables 2 and 3).

### 3.1. Systemic Administration of PDBs in Osteoporosis

**Figure 4:**
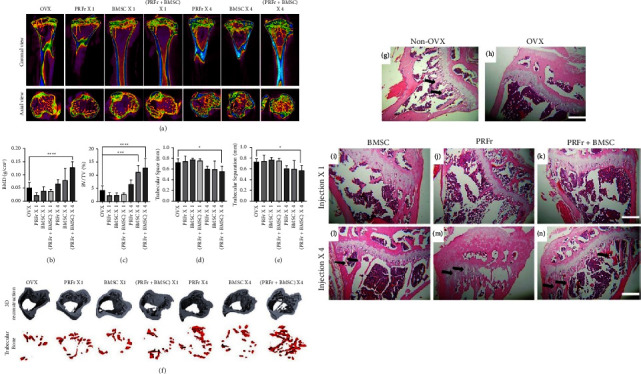
(a) Micro-CT images of ovariectomized mice in the untreated control (OVX) or experimental groups which received single/quadruple injections of either PRFr, BMSCs, or in combination therapy (PRFr + BMSCs). Comparison of a coronal and axial view of microCT images in different groups. (b–e) The BMD, bone volume versus total tissue volume (BV/TV, %), trabecular number (Tb. N), and trabecular separation (Tb. Sp) in each group of mice were evaluated 8 weeks after injection. The bars show the mean ± SD (*n* = 6) of each group. ^*∗*^*P* < 0.05; ^*∗∗∗*^*P* < 0.001; ^*∗∗∗∗*^*P* < 0.0001. (f) 3D reconstructed images of ovariectomized mice in the untreated control (OVX) or experimental groups which received single/quadruple injections of either PRFr, BMSCs, or PRFr + BMSCs (upper panel). The red region in the lower panel represents the 3D scope of the newly formed trabecular bone in the proximal tibial. (g–n) Histological sections of proximal tibial bony architecture in non-OVX mice (g) and OVX mice (h) stained by hematoxylin and eosin (H & E). (i–n) Proximal tibial sections from mice received single/quadruple injections of either PRFr, BMSCs, or PRFr + BMSCs. The black arrows indicate newly formed bony trabeculae. Scale bar: 2.5 mm. Reproduced from [22]. Copyright 2020, Springer Nature, licensed under the terms of the Creative Commons Attribution License (CC BY).

In this section, our aim is to provide a thorough evaluation of the outcomes related to the use of PDBs for osteoporosis when administered throughout the body. In this regard, Sheu et al. [23] have recently evaluated the effect of intravenous (IV) injection of PRFr, adipose-derived stem cells (ADSCs), and a combination of bon on the tibial growth plate of OVX mice. The IV injections were performed weekly for four consecutive weeks, and then the radiographical and histopathological results were obtained in the 8^th^ week after the first injection [23]. The microcomputed tomography (micro-CT) analysis showed that the sole injection of PRFr, ADSCs, or the combined application of PRFr + ADSCs significantly enhanced BMD, the bone volume to total volume (BV/TV) ratio, and the number of trabeculae inside the tibial bone which is inversely associated with the trabecular separation. No difference in the trabecular thickness was observed compared to the control group (untreated OVX mice) [23]. In all three therapeutic interventions, the serum calcium level of the rats was significantly enhanced; however, the serum phosphate level was only significantly enhanced in the group treated with PRFr + ADSCs. Thus, the authors [23] concluded that the combined application of ADSCs and PRFr was the most optimized treatment for the healing of bone defects in these osteoporotic animals. In another study by Wong et al. [22], PRFr was used in conjunction with bone marrow stem cells (BMSCs) and intravenously injected either once only or once a week for 4 consecutive weeks during the 8 week study period. PRFr alone and BMSCs alone were utilized as controls. Based on the micro-CT analysis after 8 weeks, the only group which showed significant improvements in enhancing BMD and decreasing trabecular separation and spacing was the combined application of PRFr and BMSCs (Figure 4) [22]. Both the PRFr + BMSCs and BMSCs alone groups significantly improved BV/TV while PRFr alone did not show any significant improvement which slightly contradicted the outcomes of Wong et al. [22, 23]. In their study, all groups demonstrated improvements in BV/TV following 4 weekly injections [22]. Although both Sheu et al. [23] and Wong et al. [22] performed relatively similar studies in terms of their protocols and the volume of the injection material, intervention time points, and time of sacrifice, the sole effect of PRFr was deemed more favorable in the study by Sheu et al. [23]. One noted difference between the studies was the fact that the preparation of PRFr was different between the studies which may have affected the results. Interestingly, it was observed that IV injections of ADSCs yielded better outcomes when compared to BMSCs for the treatment of osteoporosis [22, 23]; however, further clinical studies would be required to appraise these findings.

Since MSCs therapy for the treatment of osteoporosis is relatively expensive and more complicated than the utilization of platelet concentrates [37], Liu et al. [37] have assessed the sole effect of PRP injection in young and old mice using an osteoporotic model. Although the injection schedule and the volume of the injected PRP was not reported, the results indicate that the injection of PRP reversed the decreasing trend of BMD in the spine, knees, and femur [37]. Moreover, the number of trabeculae and the BV/TV ratio were significantly enhanced after 4 months [37].

In addition to the aforementioned studies evaluating the efficacy of PDBs in the treatment of osteoporosis through IV injection, one study has utilized the bone marrow transplantation approach to assess the efficacy of PDBs on osteoporosis. In this study [56], NIH3T3 embryonic fibroblasts were treated with PRP to differentiate into osteoblast-like cells. In order to assess the effect of PRP on NIH3T3 cells, four groups of the NIH3T3 cells only, PRP only, the combination of PRP/NIH3T3 cells, and a negative control group were prepared and applied for bone marrow transplantation in OVX rat models [56]. The results indicated that the combined application of PRP/NIH3T3 cells could enhance the expression of bone morphogenetic protein 2 (BMP-2) and osteopontin (OPN), resulting in reversing the bone architecture catastrophe [56]. The osteoblast-like cells migrated towards the progressing osteoporotic lesions, normalizing the bone morphology, BMD level, and trabecular architecture [56].

### 3.2. Local Administration of PDBs in Osteoporosis

In addition to the systemic administration of PDBs, their local administration has also been extensively discussed in the literature. The ensuing sections present a comprehensive evaluation of the outcomes associated with the local administration of platelet concentrates.

#### 3.2.1. Bone Regeneration for Bony Defects

The treatment of local bony defects and fractures in patients with osteoporosis can be quite challenging due to the reduced capacity of bone regeneration and weakened strength of the bone. However, the local application of PDBs has been shown to have a positive impact on bone structure, accelerating the healing process and promoting bone regeneration.

In order to manage the vertebral bone defects in osteoporosis, Cho et al. [60] evaluated the combined effect of PRP and calcium-phosphate cement (CPC) for bone regeneration in comparison to CPC alone, poly-methylmethacrylate alone, and the sham group in a caudal vertebral defect. The combination was prepared by soaking the CPC in a PRP + solvent solution in a 10 : 1 : 1 volume ratio for 5 minutes [60]. Based on the micro-CT results 2 weeks postsurgery, it was found that CPC + PRP significantly outperformed other groups in enhancing BV/TV [60]. It also exhibited significant improvements in trabecular thickness, trabecular separation, BMD, and the number of trabeculae [60]. Furthermore, based on the histological results, CPC + PRP exerted the best outcomes in bone regeneration when compared to all other groups [60]. However, the authors reported that the combination use of CPC + PRP exhibited a lower modulus when compared to the CPC group alone which might result in some shortcomings for clinical applications [60]. Correspondingly, in another study by Sakata et al. [21], the effect of PRP + gelatin/*β*-tricalcium phosphate (*β*-TCP) sponge was compared to PBS + gelatin/*β*-TCP sponge and the control group (defects with no treatment) for the treatment of an osseous defect in the third lumbar vertebral spine. The micro-CT results indicated that by the 4^th^ week, bone tissue was observed in both the PRP + gelatin/*β*-TCP sponge and the PBS + gelatin/*β*-TCP sponge groups. Nevertheless, there was a significant increase in the bone volume in the group treated with PRP compared to the group treated with PBS at 8 and 12 weeks postop (*P* < 0.05) [21]. This showed the relative long-term benefit of addition of platelet concentrates for bone regeneration when applied locally and administered with other osteoconductive biomaterials [21]. Moreover, according to the mechanical tests of the bone specimens 12 weeks postoperatively, it was found that the bone specimens treated with PRP + gelatin/*β*-TCP sponge had significantly higher stiffness (*P* < 0.05) [21]. The same results were observed regarding the compressive strength test, although the results were not statistically significant (*P* > 0.05) [21]. Amid these facts, in conclusion, the local administration of PRP for the treatment of vertebral defects resulted in some promising benefits [21, 60]. Though not seen in all studies concerning the systemic administration of PRP/PRF [22, 23, 37], future research is needed.

In another study by Engler-Pinto et al. [57], the application of either (1) leukocyte- and platelet-rich fibrin (L-PRF), (2) xenograft, or (3) L-PRF + xenograft was compared to the control group (calvaria defect filled with a blood clot) in osteoporotic rats. The comparison of L-PRF versus xenograft in osteoporotic rats indicated that runt-related transcription factor 2 (RUNX2), osteocalcin (OCN), and BMP 2/3 genes expression are equally enhanced in both groups [57]. On the other hand, vascular endothelial growth factor (VEGF) expression levels were significantly upregulated in the group treated with L-PRF [57]. This indicates that one of the main advantages of PDBs is the concomitant induction of neoangiogenesis along with osteogenesis [27]. Based on the results of the aforementioned study [57], the highest outcomes taking into consideration bone regeneration and the expression level of angio/osteogenic factors were obtained in the L-PRF + xenograft group by combining the osteoconductive properties of xenografts and enhanced bioavailability of angiogenic and osteogenic growth factors by L-PRF.

**Figure 5:**
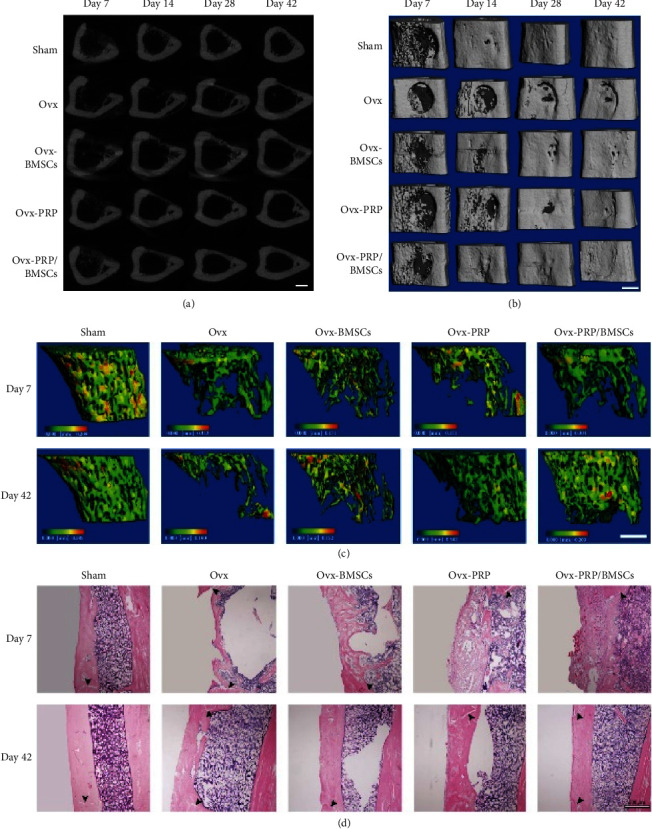
(a–c) Temporal micro-CT analysis of bone healing. Representative 2D (a) and 3D (b) images were generated by micro-CT, showing the bone healing process after drill-hole surgery. Scale bar, 1 mm. (c) Trabecular bone volumes of proximal tibial growth plates were assessed by a micro-CT scan. Scale bar, 1 mm. (d) Histological analysis of bone healing progression (stained with H & E). Representative photomicrographs of callus sections from all groups demonstrate bone healing after drill-hole surgery. Arrows indicate cortical gaps. Reproduced from [59]. Copyright 2016, Hindawi, licensed under the terms of the Creative Commons Attribution License (CC BY).

In addition, a study by Wei et al. [59] compared PRP and BMSCs to treat an osseous tibial defect either alone and combined over 42 days. The defects were filled with PBS, PRP (20 *μ*L), or/and BMSCs (1 × 10^6^ cells). Based on the micro-CT results, the only groups that showed significant improvements between the 42^nd^ and 7^th^ day postoperatively in trabecular number, trabecular separation, trabecular connectivity density, and BV/TV ratio were the groups treated with PRP-alone and PRP + BMSCs (Figure 5) [59]. According to the histological results, it was shown that by the 42^nd^ day, the thickness of each callus was similar to the adjacent cortical and lamellar bone in the group treated with PRP + BMSCs; however, there was still woven bone in the specimens treated with either PRP or BMSCs [59]. Based on the reviewed data, these combined findings demonstrate that the application of PRP + BMSCs can significantly promote bone regeneration in the defect areas among osteoporotic animals.

However, it is shown that the combined application of PDBs and MSCs may not necessarily induce synergistic effects [58]. In a study by Rocha et al. [58], a hydrolyzed collagen hemostatic sponge was used as a scaffold to deliver PRP, MSCs, or PRP + MSCs to bone defects in osteoporotic rabbits. The results of the radiographic optical densitometry of the group only treated with MSCs exhibited higher values 60 days after surgery compared to the sole application of PRP or PRP + MSCs [58]. On the other hand, the application of PRP was similar to PRP + MSCs in terms of radiographic optical densitometry [58].

#### 3.2.2. Bone Regeneration for Implant Osseointegration

Osteoporosis can also affect the bone microarchitecture which also results in more complex healing of fractures and osseous defects [66]. Since implants are one of the most predictable treatment modalities to restore function and esthetics in hard tissues [67, 68], the effect of osteoporosis on implant stability and survival rate has also been widely studied [69–74]. Unsurprisingly, osteoporosis is found to be a significant factor affecting peri-implant bone loss [71]; however, the exact mechanism of osteoporosis on implants requires a thorough investigation.

In this regard, Zhu et al. [64] have investigated the synergistic effect of dental implants with TiO_2_ nanoporous modification with PRP on implant stability in an osteoporotic rat model. Prior to implant placement, 0.1 mL/leg PRP was injected into the bone marrow cavity of the tibias of each rat, and the implants were placed in the medullary canal of the tibias [64]. The results indicated that the application of PRP with a control implant without surface modification did not enhance osseointegration; however, the sole application of dental implants with TiO_2_ nanoporous modification could significantly enhance osseointegration (*P* < 0.05). In addition, the combined application of PRP and the modified implants showed significantly better results in most of the measurements, including bone volume to total volume ratio, trabecular number, trabecular spacing, and trabecular connectivity density [64]. According to the histological results, the bone/implant contact ratio was highest for the group treated with PRP + surface-modified implants. This group also exhibited the highest volume of mature bone surrounding the implants [64]. These results indicate the potential benefits of the application of platelet concentrates during implant placement in patients diagnosed with osteoporosis [64]. Arguably, the adjunctive utilization of PDBs with standard treatment protocols could provide patients with better results and a more effective healing process.

Concerning the efficacy of PRP on the osseointegration of titanium implants, in another study by Qiao et al. [63], the lateral condyle of the distal femur of OVX rat models was used to assess the impact of two types of PRP coatings (freeze-dried and conventional) on the osseointegration of titanium implants. In in-vivo experiments, it was found that the addition of both freeze-dried and conventional types of PRP in comparison to no coating could exert significant outcomes in terms of osteogenic-related gene expression, newly regenerated BV, trabecular characteristics (thickness, number, and separation) as well as the histological and histomorphometrical assessment of the new bone [63]. Interestingly, these outcomes were significantly higher in freeze-dried PRP in comparison to PRP alone when used in conjunction with porous titanium implants in osteoporosis models [63].

Moreover, Sun et al. [62] evaluated the effect of calcium phosphate and PRP on the titanium implant osseointegration and bone regeneration of rat tibia defects. The results of BV, trabecular number, trabecular separation, and histological outcomes have indicated that the sole application of PRP accompanied with titanium has better results when compared with titanium alone (*P* < 0.05) [62]. However, the sole application of calcium phosphate-modified titanium has shown better outcomes than the sole application of PRP (*P* < 0.05) [62]. The highest outcomes in terms of BV, trabecular number, trabecular separation, and histological were obtained when PRP was used adjunctively with calcium-phosphate modified titanium implants [62]. The results indicate that the surface modification of titanium implants with calcium-phosphate may be more impactful compared to the adjunctive application of PRP in terms of bone regeneration [62, 64]. This may be attributed to the longer effect of calcium phosphate compared to the fast degradation rate of PRP [29, 62, 64].

Among the aforementioned studies assessing the impact of PDBs on osseointegration [62–64], no study has assessed PRF [62]. In this context, Omar et al. [61] have assessed the combined effect of local administration of PRF and the systemic administration of calcitonin on the osseointegration of tibia implants. This study consisted of three groups: implant alone, implant combined with calcitonin, and implant with the combined application of calcitonin and PRF in the tibia defect of osteoporotic rabbit models [61]. Twelve weeks after insertion of implants, the animals were euthanized, and the specimens were evaluated in terms of the width of the gap between bone and the implant threads as well as the percentage of the implant area covered with bone [61]. The results indicated that the group containing both calcitonin and PRF (0.63 ± 0.005 *μ*m) had significantly less gap width compared to the sole application of calcitonin with implants (1.85 ± 0.52 *μ*m) and the group of implants alone (5.98 ± 0.74 *μ*m) (*P* < 0.001). Moreover, upon assessment of the percentage of implant surface area covered with bone, it was similarly demonstrated that the group containing both calcitonin and PRF (95.68 ± 2.7%) had significantly higher values compared to the sole application of calcitonin with implants (54.26 ± 4.1%) and the group of implants alone (21.76 ± 4.8%) (*P* < 0.001). The authors have concluded that the combined application of the local PRF and systemic calcitonin can be an effective technique to accelerate and enhance bone regeneration and osseointegration around bone implants [61]. The variables assessed by the study conducted by Omar et al. [61] were more clinically practical to assess the level of implant surface osseointegration compared to the previous studies [62–64].

## 4. Rationale for Selection between Systemic or Local Administration of PDBs

As mentioned previously, numerous studies have explored the use of systemic and local administration of PDBs [21–23, 37, 56–64]. However, what are the considerations and rationales guiding the choice between systemic or local utilization of these biomaterials?

The systemic administration of platelet concentrates has demonstrated potential for enhancing bone microstructure in a generalized manner, leading to improved bone healing and regeneration throughout the skeletal system. Consideration may be given to systemic administration as a supplementary therapy to promote overall skeletal health. On the other hand, local administration of platelet concentrates has shown notable benefits for osteoporotic bone structures. It promotes bone regeneration and osseointegration in proximity to bone implants, while also stimulating osteogenic differentiation and counteracting the propensity toward adipogenic differentiation within the bone structure. The utilization of local administration can be contemplated as an adjunct treatment option for targeted enhancement of bone regeneration and osseointegration.

Given the extent of the disease and patient-specific factors, the choice between local and systemic administration of PDBs should be carefully considered. Further investigation is needed to better understand the nuances associated with each approach, to assess the magnitude of their effects, and to achieve more conclusive results.

## 5. Molecular Mechanism of PDBs in Osteoporosis

**Figure 6:**
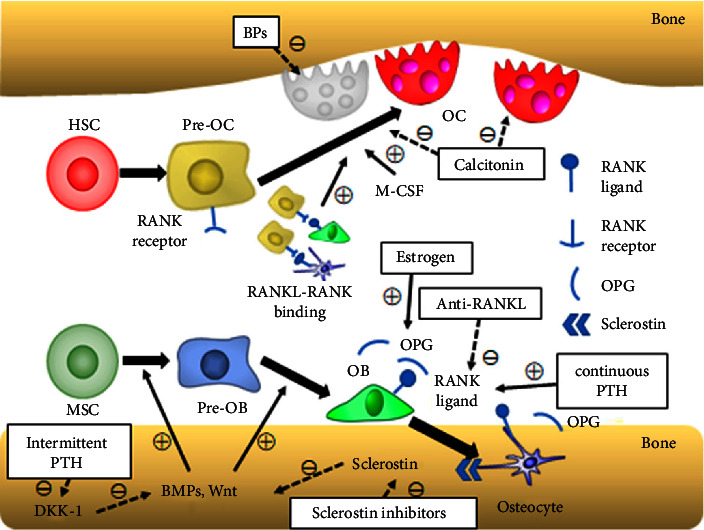
Schematic diagram of bone homeostasis and a summary of the action mechanism of the agents for osteoporosis. Normal arrows with “+” mean a positive effect; dotted arrows with “−” mean a negative effect. Reproduced from [76]. Copyright 2019, MDPI, licensed under the terms of the Creative Commons Attribution License (CC BY).

Bone hemostasis or remodeling is a continuous process in which new bone tissue is formed by osteoblasts through bone formation, and mature bone tissue is broken down by osteoclasts through bone resorption [75] (Figure 6). Osteoblasts, which are responsible for bone formation, originate from MSCs. The transcription factor RUNX2 is crucial for osteoblast differentiation, and its expression stimulates MSCs to become osteoblasts. RUNX2 is regulated by signals such as BMPs and the Wnt/*β*-catenin pathway. BMPs activate RUNX2 through phosphorylation of SMAD1/5/8, while Wnt proteins increase RUNX2 levels via *β*-catenin stabilization or protein kinase C*δ* [76]. On the other hand, osteoclasts, which are responsible for bone resorption, differentiate from hematopoietic stem cells in response to monocyte/macrophage colony-stimulating factor (M-CSF) and RANKL stimulation [76]. RANKL, produced by osteoblasts and osteocytes, binds to RANK on osteoclast precursor cells, leading to their differentiation into osteoclasts. Osteoprotegerin (OPG) which is also produced by osteoblasts prevents RANKL binding to RANK and influences the regulation of osteoclast activity. The interaction between RANKL, RANK, and OPG is essential for maintaining bone homeostasis by regulating osteoclast function [76].

In contrast to bone hemostasis, osteoporosis is caused by an imbalance in the bone remodeling process. Osteoporosis leads to a decrease in the secretion of OPG by osteoblasts and an increase in the expression and secretion of RANKL, interleukin 1(IL-1), IL-6, IL-11, and tumor necrosis factor *α* (TNF-*α*). These compounds directly stimulate greater formation and activity of osteoclasts. The reduced levels of OPG also allow for stronger binding of RANKL to RANK, further facilitating increased osteoclastogenesis and bone resorption [77]. In addition, in osteoporotic patients, BMSCs have a reduced ability to differentiate into osteoblasts and an increased tendency to differentiate into adipocytes. This dual effect contributes to a further decrease in bone formation and an increase in the accumulation of fat within the bone marrow. In this process, PPAR-*γ* plays a crucial role by promoting adipogenic differentiation of BMSCs by regulating the expression of adipogenic genes [78].

According to the existing evidence, PDBs seem to reverse osteoporosis by enhancing the number of osteoblast-like cells and inducing osteogenic differentiation while inhibiting adipogenic differentiation [37]. The trans-differentiation of adipocytes to osteoblasts by platelet concentrates is performed by gene knockout of PPAR-*γ* and leptin which are indicators of adipogenic differentiation. On the other hand, the transcriptional and translational markers of osteogenic differentiation (RUNX2, OPN, and OCN) enhance due to the presence of platelet concentrates [37]. In addition, RANKL, an osteoclast bone resorption factor, seems to have decreased in the presence of platelet concentrates [37]. PDBs also encompass a wide variety of growth factors [79], including platelet-derived growth factor (PDGF), vascular endothelial growth factor (VEGF), insulin-like growth factor (IGF), and transforming growth factor-*β* (TGF-*β*). It has been demonstrated that PDGF can induce osteoblast proliferation and differentiation [37, 80]. Moreover, VEGF can enhance neoangiogenesis and its ability to activate BMP signaling pathways that can further enhance osteogenic differentiation [81]. Furthermore, TGF-*β*1 has been shown to have inhibitory effects on adipogenic differentiation while also exhibiting positive effects on osteogenic differentiation [37, 82–84]. IGF seems to be an important compound in the synthesis of bone matrix and the elimination of fat tissue [37, 85]. In a study conducted by Liu et al. [37], 3T3-L1 cells (mouse preadipocytes) were treated with PRP to see whether their phenotypes would be modified towards osteogenic differentiation. They found that the presence of PRP enhanced the dynamic expression of BMP-2 and its receptor (BMPR) [37]. On the other hand, another isoform of BMPR-IB is BMPR-IA which is a receptor for BMP-2 for adipogenic differentiation and is deactivated by BMP-2 [37]. This process explains why platelet concentrates have the potential to simultaneously enhance osteoblastic differentiation and inhibit adipogenic differentiation [37].

The potential therapeutic effects of platelet concentrates specifically in osteoporotic patients can also be explained by the fact that platelets' function and morphology tend to exhibit deviations from normal function in the population of people with osteoporosis [86]. The evaluation of serum platelets in osteoporotic patients has shown interesting results [86]. Concerning the function and morphology of serum platelets mean platelet volume (MPV) [87, 88] and platelet distribution width (PDW) [87] in osteoporotic patients, it was shown that the aforementioned factors are associated with BMD and tend to decrease in patients with osteoporosis. It has also been demonstrated that the platelet-to-lymphocyte ratio is associated with low BMD, especially in the femoral and lumbar parts which are key areas in determining and measuring BMD during osteoporosis diagnosis [89, 90]. What is more, platelet-activating factor (PAF) may affect platelet function, leading to an increased risk of osteoporosis. The assessment of the serum concentration of PAF in osteoporotic women has demonstrated that a low PAF serum level is associated with an increased presence of vertebral fractures and lower BMD [91].

Based on the evidence concerning the importance of platelet function and morphology in patients with osteoporosis [86], it can be postulated that these platelets alterations associated with the aging process can yield gradual deterioration in osteoporotic patients.

## 6. Comparison of PDBs to Standard Treatments in Osteoporosis

To evaluate the suitability of PDBs for treating osteoporosis, it is important to compare their efficacy, safety, and adverse effects with the established pharmacological treatment approaches currently used. Since bisphosphonates are widely used as the standard treatment options for osteoporosis in clinical practice [92], we have selected them for such a comparison. This assessment will provide insights into the applicability of PDBs and aid in determining their potential benefits and limitations for osteoporosis treatment.

Regardless of their safety and adverse effects, bisphosphonates have been extensively studied and proven to effectively reduce the risk of bone fractures. However, their efficacy can vary depending on various factors such as bone density, age, and other individual risk factors [93]. On the other hand, numerous studies have demonstrated the positive effectiveness of PDBs in promoting bone tissue regeneration [94]. PDBs contain high levels of growth factors and regenerative cells, enabling them to stimulate osteogenic differentiation and inhibit adipogenic differentiation [37, 95]. However, there is still limited research specifically investigating the effects of PDBs on osteoporosis. Additionally, there is a lack of studies directly comparing the efficacy of PDBs with bisphosphonates or other treatment options for osteoporosis. As a result, it is challenging to make a direct comparison regarding their effectiveness.

In terms of safety and adverse effects, PDBs are generally considered safe and have a low risk of adverse effects. This is primarily due to their autologous nature, which reduces the chances of immune reactions [26, 47]. However, since the procedure for obtaining PDBs involves a blood draw, patients should make sure they are well hydrated and have eaten beforehand to prevent feeling lightheaded. It's also crucial to consider the potential side effects associated with the administration of PDBs. These may include minor complications like bleeding, tissue damage, infection, and nerve injury. Unlike PDBs administration, it's important to note that there can be some severe complications associated with bisphosphonate treatment due to their chemical nature. These potential complications may include gastrointestinal side effects, acute phase response, renal failure, osteonecrosis of the jaw, atypical femoral fractures, atrial fibrillation and cardiovascular risk, musculoskeletal pain, ocular pain, and cutaneous manifestations [96]. It should also be noted that these adverse effects may vary depending on whether the medication is taken orally as tablets or given intravenously through infusion [97].

## 7. Limitations and Future Prospects

Until now, no study has comprehensively reviewed platelet concentrates and their effect on osteoporotic defects. Undoubtedly, outcomes from these studies decisively suggest the beneficial effects of platelet concentrates to minimize the detrimental impact of osteoporosis and also improve the healing process of bone [21–23, 37, 56–64]. However, this paper heavily relies on in vitro and in vivo experiments to support the efficacy of PDBs in osteoporosis treatment. While these studies provide valuable insights, the absence of substantial clinical evidence involving human subjects is a major concern, and the extrapolation of animal results to human applications needs to be addressed with caution. Therefore, it is essential to conduct future randomized controlled trials (RCTs) to evaluate the efficacy, safety, and cost-effectiveness of platelet concentrates in comparison to conventional treatment options for patients diagnosed with osteoporosis, especially those with comorbidities. These RCTs should also take into consideration risk factors such as age and polypharmacy. By conducting such studies, we can obtain more substantial evidence to determine the potential benefits and drawbacks of platelet concentrates in clinical applications for osteoporosis treatment.

In addition, outcomes from the administration of the new generation of liquid platelet concentrates called I-PRF have shown promising outcomes in regenerative medicine, especially bone tissue regeneration [22, 23, 37]. In this regard, the authors postulate that the application of I-PRF would further enhance the beneficial effects of platelet concentrates when compared to PRP or PRFr since I-PRF is a richer source of growth factors [29, 55]. Moreover, since the duration of growth factors released from platelet concentrates is dependent upon their degradation time, new attempts have been made to prolong their degradation time [98, 99]. In this regard, crosslinking with carbodiimide [98] or utilization of a new generation PRF named albumin gel-PRF (Alb-PRF) [99] has provided new opportunities for these biomaterials to be utilized over extended periods. Therefore, future studies can focus on the administration of I-PRF and Alb-PRF for the treatment of osteoporosis in in vitro and in vivo environments.

Furthermore, this review article showed that many more studies were conducted on the local administration of PDBs in comparison to their systemic administration. Even though the local administration of platelet concentrates has shown positive results in all the studies [21, 57–64], it is important to note that such effects were merely limited to the defect area, while their systemic administration may have potential general benefits within the skeletal system [22, 23, 37, 56]. Hence, further studies are required to be conducted on the systemic application of PDBs to prove whether they are truly beneficial for the skeletal system.

## 8. Conclusion

Considering the limitations of this review, it can be concluded that both the local and systemic administration of platelet concentrates can have a beneficial effect on osteoporotic bone structures. However, the magnitude of such an effect and the choice of systemic versus local administration of platelet concentrates are dependent upon the vastness of the disease. Based on the molecular and cellular mechanisms regarding the effects of platelet concentrates on osteoporosis, arguably, PDBs can reverse adipogenic differentiation towards osteogenic differentiation. Although the results of the existing studies seem promising, it must be emphasized that all studies to date were conducted using in vitro and nonhuman in vivo models. Therefore, further future clinical studies are needed to ensure the clinical efficacy of platelet concentrates in patients diagnosed with osteoporosis.

## Figures and Tables

**Table 1:** Summary of the differences between PRP and PRF [45, 53].

PDBs	Platelet-rich plasma (PRP)	Platelet-rich fibrin (PRF)
**Type of additive in the blood sample**	Anticoagulant (bovine thrombin/calcium chloride)	None
**Fibrin polymerization rate**	Rapid (dependent upon the amount of anticoagulant)	Gradual (natural polymerization in the glass/blood contact area)
**Quality of the fibrin network**	Rigid network (condensed tetra-molecular/bilateral junctions due to high anticoagulant content)	Flexible network (trimolecular or equilateral junctions)
**Effect of fibrin network on cell migration and cytokine release**	Unfavorable (due to network rigidity)	Favorable (due to network flexibility)
**Modulus of elasticity of the fibrin network**	High (due to network rigidity)	Low (due to network flexibility)

**Table 2:** Summary of the studies on systemic administration of PDBs.

Authors (Year)	Animal model	Study Group(s)	Control group	Preparation protocol of PDBs	The application method of PDBs	Method of evaluation	Main outcomes	Follow-up	References
Sheu et al. (2020)	OVX mice	I: ADSCs II: PRFr III: ADSCs + PRFr	I: Unoperated mice II: Operated non-OVX mice III: Operated OVX mice	6 ml blood centrifuged at: 3000 g in 10 min in order to obtain PRF PRF was centrifuged at: 5000 g in 10 min to obtain PRFr	0.6 mL IV injection/week for 4 weeks	I: micro-CT II: Histology III: Serum Ca and phosphate evaluation	OVX—ADSCs + PRFr exerted the best results in bone consolidation and bone tissue production	8 weeks after the 1^st^ injection	[23]

Wong et al. (2020)	OVX mice	I: PRFr (1×) II: BMSCs (1×) III: PRFr + BMSCs (1×) IV: PRFr (4×) V: BMSCs (4×) VI: PRFr + BMSCs (4×)	Unoperated OVX mice	8 ml blood centrifuged at: 400 g in 10 min in order to obtain PRF PRF was centrifuged at: 3000 g in 10 min to obtain PRFr	0.6 mL IV injection for the whole study period/1 IV injection/week for 4 weeks	I: Flow cytometry analysis II: Osteogenic differentiation assay III: micro-CT IV: Histology	Groups with single injection didn't exhibit any significant results. However, in the groups with 4 injections, PRFr + BMSCs showed the highest outcomes	8 weeks after the 1^st^ injection	[22]

Liu et al. (2011)	OVX mice	I: Young OVX mice + PRP + PBS (1 month-old) II: Old OVX mice + PRP + PBS (10 months-old)	I: Young OVX mice with PBS injection (1 month-old) II: Old OVX mice with PBS injection (10 months-old)	NR	Inside bone marrow cavity injection	I: Immunohistochemistry II: BMD and micro-CT III: Gene expression IV: Western blot	PRP + PBS inhibited adipocyte differentiation and induced osteogenic differentiation in adipocytes. Moreover, PRP + PBS induced bone regeneration and avoided a further bone loss in osteoporotic mice	4 months postoperatively	[37]

Lo et al., (2009)	OVX mice	I: NIH3T3 cells II: PRP III: PRP/NIH3T3 cells	Sham group	Blood centrifuged at 3000 g in 6 min	1% PRP was applied to the calf serum containing DMEM	I: Osteogenic differentiation II: RT-PCR III: In vivo fluorescence imaging IV: BMD assessment V: Ultrastructural analysis VI: IHC VII: Western blot technique	The bone marrow transplantation of PRP-conditioned NIH3T3 cells prolonged the life span of the OVX rats and enhanced the bone quality	0, 1, 2, and the 3 months postoperatively	[56]

ADSCs, adipose-derived stem cells; BMD, bone mineral density; BMSCs, bone marrow stem cells, IV: intravenous, microCT, microcomputed tomography; OVX, ovariectomized; PBS, phosphate‐buffered saline, PRFr, PRF releasate; PRP, platelet-rich plasma; NR, not reported.

**Table 3:** Summary of the studies on local administration of PDBs.

Authors (Year)	Animal model	Study Group(s)	Control group	Preparation protocol of PDBs	The application method of PDBs	Method of evaluation		Main outcomes	Follow-up	References
*Bone regeneration for bony defects*										
Engler-pinto et al. (2019)	OVX rats	I: PRF II: Xenograft III: PRF + xenograft	Unfilled artificial defects	3.5 ml blood centrifuged at 2700 g in 12 min	0.1 mL of materials inserted into an artificial calvaria defect	I: micro-CT II: Histology III: Immunohistochemistry	PRP + xenograft exhibited the best outcomes in bone formation and upregulated osteogenic gene expression		4 months postoperatively	[57]
Rocha et al., (2017)	OVX rabbits	I: PRP + collagen sponge II: MSCs + collagen sponge III: PRP + MSCs + collagen sponge	Collagen sponge	Blood was added to calcium gluconate and centrifuged at 1500 rpm for 4 min	PRP with the cellular content of 1 × 10^6^ platelets loaded on 3 mm fragments of collagen sponge artificial tibia defect	I: Radiographic optical densitometry II: Histology	The sole application of MSCs exerted better outcomes compared to PRP or PRP + MSCs groups		30 and 60 days postoperatively	[58]
Sakata et al. (2017)	OVX rats	I: PRP + gelatin + *β*-TCP II: PBS + gelatin + *β*-TCP	Unfilled artificial defects	8 ml blood + 2 ml EDT centrifuged at: 2000 g in 10 min Followed by: 1000 g in 15 min	Materials implanted into lumbar vertebral body defect	I: micro-CT II: Histology III: Biomechanical testing	PRP + gelatin + *β*-TCP induced statistically significant bone regeneration (*P* < 0.05) and it also exerted significantly higher stiffness (*P* < 0.05)		4, 8, and 12 weeks postoperatively	[21]
Wei et al. (2016)	OVX rats	I: PRP II: BMSCs III: PRP + BMSCs	I: Non-OVX rats receiving PBS II: OVX rats receiving PBS	Blood + heparin centrifuged at: 215 g in 10 min Followed by: 863 g in 10 min at 20°C	Material implantation in an artificial defect in the tibia	I: microCT II: Histology III: Gene expression	Groups treated with PRP and PRP + BMSCs exhibited the best outcomes in bone regeneration and osteogenic gene upregulation		42 days postoperatively	[59]
Cho et al. (2014)	OVX rats	I: Poly-methylmethacrylate II: CPC III: CPC + PRP	Unfilled artificial defects	8 ml blood + EDTA saline centrifuged at: 200 g in 10 min Plasma portion centrifuged at: 400 g in 15 min	Material implantation in an artificial defect in caudal vertebral body	I: micro-CT II: Histology	Higher trabecular bone volume fraction, trabecular thickness, BMD, and overall bone regeneration		2 weeks postoperatively	[60]

*Bone regeneration for implant osseointegration*										
Omar et al., (2021)	OVX rabbits	I: Implant + calcitonin II: Implant + calcitonin + PRF	Implant without PRF and calcitonin	8 mL centrifuged at 3000 rpm for 10 min	PRF implanted in the osteotomized tibia site before implant placement	I: SEM II: EDX		The combined application of implant + calcitonin + PRF resulted in high bone-to-implant contact and less gap between the implant and the bone	12 weeks postoperatively	[61]
Sun et al., (2021)	OVX rats	I: Implant + CaP II: Implant + PRP III: Implant + CaP + PRP	Implant without PRP	16 mL blood centrifuged at 180 g for 10 min Followed by: Centrifugation at 1000 g for 10 min	PRP injected into the bone marrow cavity of tibia followed by implants positioning in the tibia medullary canal	I: micro-CT II: Biomechanical test III: Histology		The combined application of implant + PRP + CaP resulted in the highest outcomes in terms of implant stabilization	12 weeks postoperatively	[62]
Qiao et al., (2020)	OVX rabbits	I: 3D—printed pTi + PRP II: 3D—printed pTi + freeze-dried PRP	3D—printed pTi implants	5 mL blood centrifuged at 209 g for 16 min Followed by centrifugation at 1500 g for 12 min	pTi was immersed in PRP for 5 min followed by the addition of thrombin and CaCl_2_	I: Cell viability II: Osteogenic differentiation III: micro-CT IV: Histology		The coating of freeze-dried PRP showed superior cell activity and osteogenic potential compared to conventional PRP	6 and 12 weeks postoperatively	[63]
Zhu et al. (2016)	OVX rats	I: TiO_2_ implant II: Control implant + PRP III: TiO_2_ implant + PRP	Unfilled artificial defects	Blood centrifuged at: 180 g in 10 min Followed by: 1000 g in 10 min [65]	Implant insertion and PRP injection inside the tibia bone marrow cavity	I: Field-emission SEM II: AFM III: XRD IV: micro-CT V: Histology VI: Biomechanical testing VII: SEM		PRP could enhance osteogenesis earlier than TiO_2_ implant; however, the best outcomes were achieved in PRP + TiO_2_ implant group	12 weeks postoperatively	[64]

ADSCs, adipose-derived stem cell; AFM, atomic force microscope, BMD, bone mineral density; BMSCs, bone marrow stem cell; *β*-TCP, *β*-Tri calcium phosphate; DMEM, Dulbecco's modified Eagle's medium; EDX, energy-dispersive X-ray spectroscopy; IV, intravenous; micro-CT, microcomputed tomography; MSCs, Mesenchymal stem cells; OVX, ovariectomized; PBS, phosphate‐buffered saline; PRFr, PRF releasate; PRP, platelet-rich plasma; pTi, porous titanium, SEM, scanning electron microscope; XRD, X-ray diffraction.

## Data Availability

No underlying data were collected or produced in this study.
